# Improving the efficiency of dye-sensitized solar cells based on rare-earth metal modified bismuth ferrites

**DOI:** 10.1038/s41598-023-30000-8

**Published:** 2023-02-22

**Authors:** Maham Khan, Muhammad Aamir Iqbal, Maria Malik, Syed Usama Mauood Hashmi, Sunila Bakhsh, Muhammad Sohail, Muhammad Tariq Qamar, Mohammed Al-Bahrani, Rey Y. Capangpangan, Arnold C. Alguno, Jeong Ryeol Choi

**Affiliations:** 1 Department of Chemistry, Forman Christian College, Lahore, 54600 Pakistan; 2 School of Materials Science and Engineering, Zhejiang University, Hangzhou, 310027 China; 3 Centre of Excellence in Solid State Physics, University of the Punjab, Lahore, 54590 Pakistan; 4 Department of Physics, Balochistan University of Information Technology, Engineering and Management Sciences, Quetta, 87300 Pakistan; 5 Department of Physics, University of Balochistan, Quetta, 87300 Pakistan; 6 Chemical Engineering and Petroleum Industries Department, Al-Mustaqbal University College, Babylon, 51001 Iraq; 7 Department of Physical Sciences and Mathematics, College of Marine and Allied Sciences, Mindanao State University at Naawan, Poblacion, 9023 Naawan, Misamis Oriental Philippines; 8 Department of Physics, Premier Research Institute of Science and Mathematics (PRISM), Mindanao State University - Iligan Institute of Technology, Tibanga Highway, 9200 Iligan City, Philippines; 9 School of Electronic Engineering, Kyonggi University, Suwon, Gyeonggi-do 16227 Republic of Korea

**Keywords:** Biophysics, Energy science and technology, Materials science, Physics

## Abstract

This study reports light energy harvesting characteristics of bismuth ferrite (BiFeO_3_) and BiFO_3_ doped with rare-earth metals such as neodymium (Nd), praseodymium (Pr), and gadolinium (Gd) dye solutions that were prepared by using the co-precipitation method. The structural, morphological, and optical properties of synthesized materials were studied, confirming that 5–50 nm sized synthesized particles have a well-developed and non-uniform grain size due to their amorphous nature. Moreover, the peaks of photoelectron emission for bare and doped BiFeO_3_ were observed in the visible region at around 490 nm, while the emission intensity of bare BiFeO_3_ was noticed to be lower than that of doped materials. Photoanodes were prepared with the paste of the synthesized sample and then assembled to make a solar cell. The natural and synthetic dye solutions of *Mentha, Actinidia deliciosa*, and green malachite, respectively, were prepared in which the photoanodes were immersed to analyze the photoconversion efficiency of the assembled dye-synthesized solar cells. The power conversion efficiency of fabricated DSSCs, which was confirmed from the I–V curve, is in the range from 0.84 to 2.15%. This study confirms that mint (*Mentha*) dye and Nd-doped BiFeO_3_ materials were found to be the most efficient sensitizer and photoanode materials among all the sensitizers and photoanodes tested.

## Introduction

The current sources of energy include fossil fuels like coal, oil, and natural gas, but the situation of their usage is becoming alarming. This shortcoming of energy has been answered now by the efficient production of solar energy using advanced photovoltaic technologies in connection with dye-sensitized solar cells (DSSCs)^1–3^. The applications of dye-sensitized solar cells are found to be very significant in up-to-date scientific branches like wireless sensor networks (smart buildings, smart homes, and smart cities), medical devices, sports, security sensors, cameras, and wearable electronics^4^. A DSSC mainly converts photons present in sunlight into electrical energy. The four key parameters of a dye-sensitized solar cell are the working electrode, sensitizer (dye), redox-mediator (electrolyte), and counter electrode, wherein an electrolyte system for a redox reaction can be coupled and sandwiched between two glass plates of the photoanode and counter electrode. The DSSC's photoconversion efficiency (PCE) depends on the efficiency of each individual component involved, and the photoanode plays a key role in the charge generation and transfer processes. The photoanode is a glass plate made of a thin layer of conductive oxide, and likewise, the counter electrode is made of the same material with an additional coating of another catalytic material.

In previous DSSCs studies, several semiconductors were reported to be employed as photoanodes, including transition metal oxides such as TiO_2_, ZnO, SnO_2_, and Nb_2_O_2_, whose disadvantages outweighed their advantages^5,6^. TiO_2_ has traditionally been used as a catalyst in the synthesis of photoanodes, but its wide bandgap absorbs only UV light and is susceptible to photocarrier recombination, resulting in a low utilization of the visible light spectrum^7^. Therefore, alternative materials are being studied recently to overcome the existing limitations, wherein perovskite semiconductors, such as BaTiO_3_, LiNbO_3_, and BiFeO_3_, are getting much attention, thanks to their appropriate bandgap for UV to visible light sensitization^8,9^. Among these, ferroelectric materials are reported to have more potential in photovoltaic activity in relation with the production of photoexcited charge carriers that can generate a polarization-induced electric field which induces dispersion of these photogenerated charge carriers^10^. Bismuth ferrite (BiFO_3_) with a narrow bandgap of 2.68 eV is considered as a high-performance light catalyst with a unique twisted rhombohedral perovskite structure. Such a structure reduces the photocarrier recombination and supports the carrier transmission, resulting in improvement of the utilization of visible light spectra^11–13^. The large surface area of bismuth ferrite (BFO) nanoparticles efficiently absorbs dye molecules for radiant energy harvesting, anchoring the carboxylic acid, hydroxyl, and carbonyl functional groups associated with the dye material, which promotes high electron injection into the conduction band. Energy harvesting can also be improved by enhancing their electron transfer and boding with dye sensitizers via the doping of rare-earth materials^14^.

However, to get a high efficiency of light harvesting with DSSC, the most adopted strategy is to make the bandgap of the active material narrower by forming a heterojunction with the doping of another material, which leads to improvement of the light absorption range^15,16^. An’amt et al. used bismuth–titanium dioxide (Bi–TiO_2_) nano-cubes as photoanodes for the fabrication of DSSCs, whose power conversion efficiency (η) was found to be enhanced by 2.11%^17^. However, dye materials also have an impact on improving the efficiency of DSSC by absorbing photon energy and excitably producing energy on their de-excitation. Transition metals with high charge transfer abilities, such as ruthenium carboxylated polypyridyl complexes, are currently used as dyes^18^. Currently, natural dyes are replacing these complex molecules due to their economical, eco-friendly, and simple methodology with efficient results^19^, displaying a large absorption coefficient due to π to π^*^ transition and the high number of natural functional groups available. Natural dyes obtained from plants have been used as sensitizers to improve the efficiency of DSSCs. Natural dyes that we use should have a robust and broad capacity in absorbing light in the visible and near-IR regions.

Wegene fabricated DSSCs using a photoanode of ZnO and natural dyes, such as beetroot, Amaranthus Iresine Herbstii, Bougainvillea spectacles, and flowers. The highest power conversion efficiency was found to be 0.03%^20^. Likewise, Nirmala et al. used nanocrystalline titanium dioxide (TiO_2_) as a photoanode coated with FTO and anchored into 10 natural dye extracts separately, such as beetroot, mint, blueberry, and turmeric. The highest power conversion efficiency was found to be 0.03% in the case of blueberry dye^21^. While doping of the active material and treating it with natural dye improved the efficiency of DSSC, as reported by Prabu et al. who fabricated DSSCs using different photoanodes, namely, Mg-doped TiO_2_, Al-doped TiO_2_, Bi-doped TiO_2_, and ZnO-doped TiO_2_ sensitized with natural dyes, namely, Rama Tulsi (Ocimum sanctum)^22^. The efficiencies of DSSC for Zn-doped TiO_2_, Al-doped TiO_2_, Bi-doped TiO_2_, and Mg-doped TiO_2_ were reported to be 0.65%, 0.59%, 0.48%, and 0.54%, respectively^17–22^. The high performance of natural dye materials, including plant parts such as leaves, flowers, fruit, pulp, and root pulp, was observed in improving the DSSC efficiency, involving natural chemicals such as xanthene, anthocynin, carotenoid, chlorophyll, flavonoid, and carboxylic acid^18,23^. This results in high electron transfer and combination and hence improves the efficiency of DSSCs^24^.

This study is aimed at fabricating high-performance DSSCs based on BFO perovskite-doped rare earth metals, including Nd, Gd, and Pr, with the natural dye sensitizers Mentha and Actinidia deliciosa along with the synthetic green malachite dye. The efficiency of fabricated DSSCs is relatively high compared to the pristine BFO-based DSSC when doped with rare earth metals. The efficiency has been improved from 0.84 to 2.15%, and this confirms the novelty of this work. Results have proven that PCE is higher for the doped BFO photoanodic material. Hence, efficient photoanodic materials can be prepared by utilizing green synthesis methods, which are considered to be non-toxic and cost-effective, and this method is an initial attempt to get an efficient solar device at a low manufacturing cost.

## Materials and method

All the reagents used in this research were of analytical grade and utilized without additional purification. The purity of malachite green was 88–90%, ethanol 96%, potassium iodide 95%, sulphuric acid 98%, potassium hydroxide 85%, bismuth (III) nitrate pentahydrate 98%, and iron (III) nitrate 98%, whereas each of the gadolinium (III) nitrate hexahydrate, neodymium (III) nitrate hexahydrate, and presodymium (III) nitrate hexahydrate had 99.9% purity. All these materials were obtained from Sigma-Aldrich and Merck (distributors from Lahore, Pakistan), except the potassium iodide, which was obtained from Supelco Company, Lahore, Pakistan.

### Synthesis of BiFeO_3_

**Figure 1:**
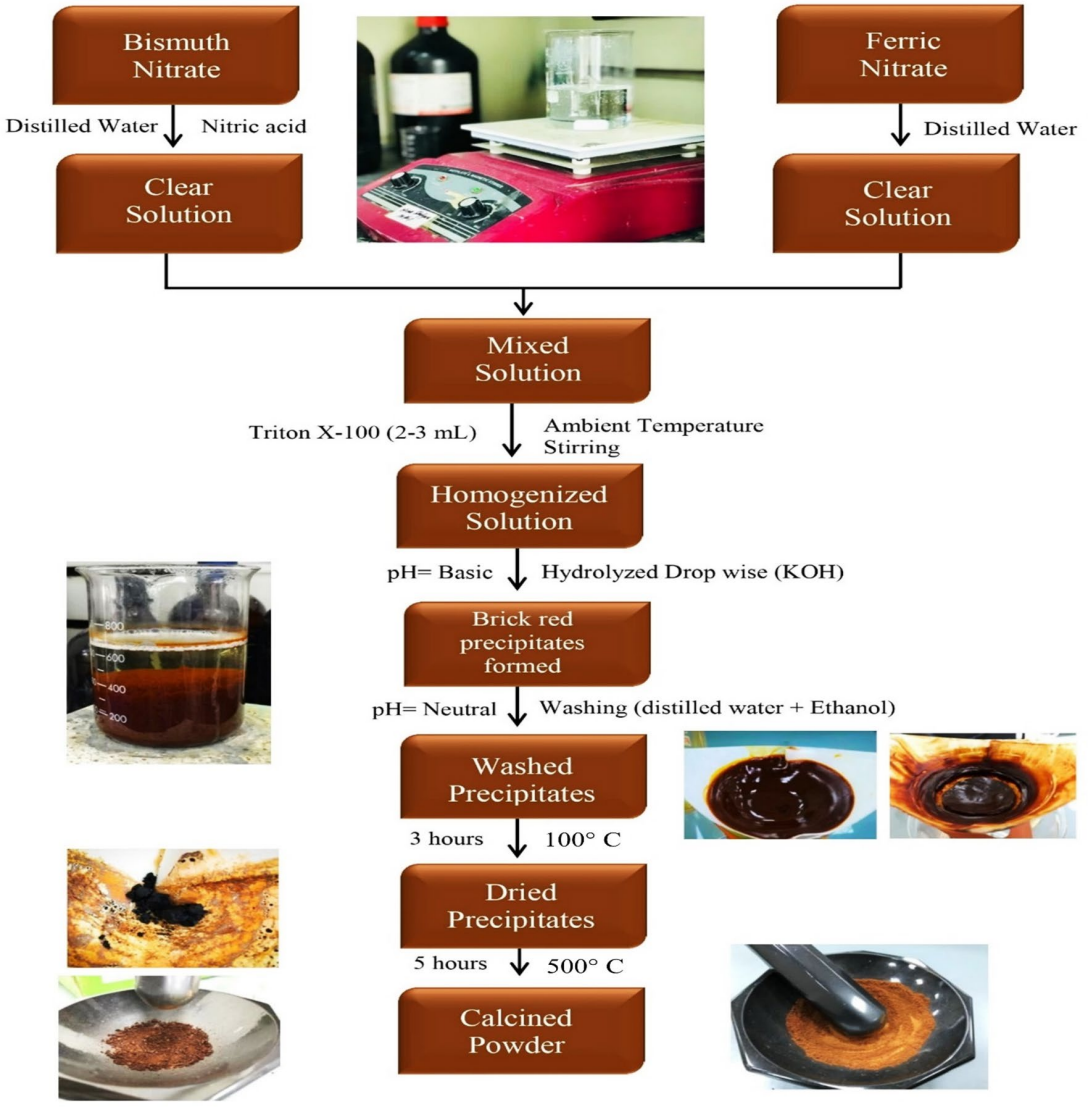
Synthesis process of BiFeO_3_.

A pristine BiFeO_3_ photoanode was created using a standard synthesis method as shown in Fig. 1. At first, we dissolved 2.32 g of bismuth (III) nitrate pentahydrate (Bi(NO_3_)_3_·5H_2_O) in 50 mL of distilled water compounded with 15 mL of concentrated nitric acid (HNO_3_). In a separate beaker, 7.23 g of iron (III) nitrate (Fe(NO_3_)_3_·9H_2_O) was dissolved in 50 mL of distilled water. Both of these solutions were mixed slowly, followed by the addition of 2 mL of Triton X-100 with continuous stirring. The mixture was allowed to heat at ambient temperature with continuous stirring for 2 h. Then the mixture was hydrolysed slowly with 3 M potassium hydroxide (KOH) using a burette until the pH of the solution became basic and brick-red precipitates were formed. The precipitates were washed with distilled water until the pH became neutral. Later on, the precipitates were also washed twice with 20 mL of ethanol–water solution. Precipitates were dried in an oven at 100 °C for 4 h and calcined at 500 °C for 4 h. The calcined material was again subjected to a grinding process by using a mortar and pestle, then collected in a vial and subjected to further characterization.

### Synthesis of rare earth metals doped BiFeO_3_

**Figure 2:**
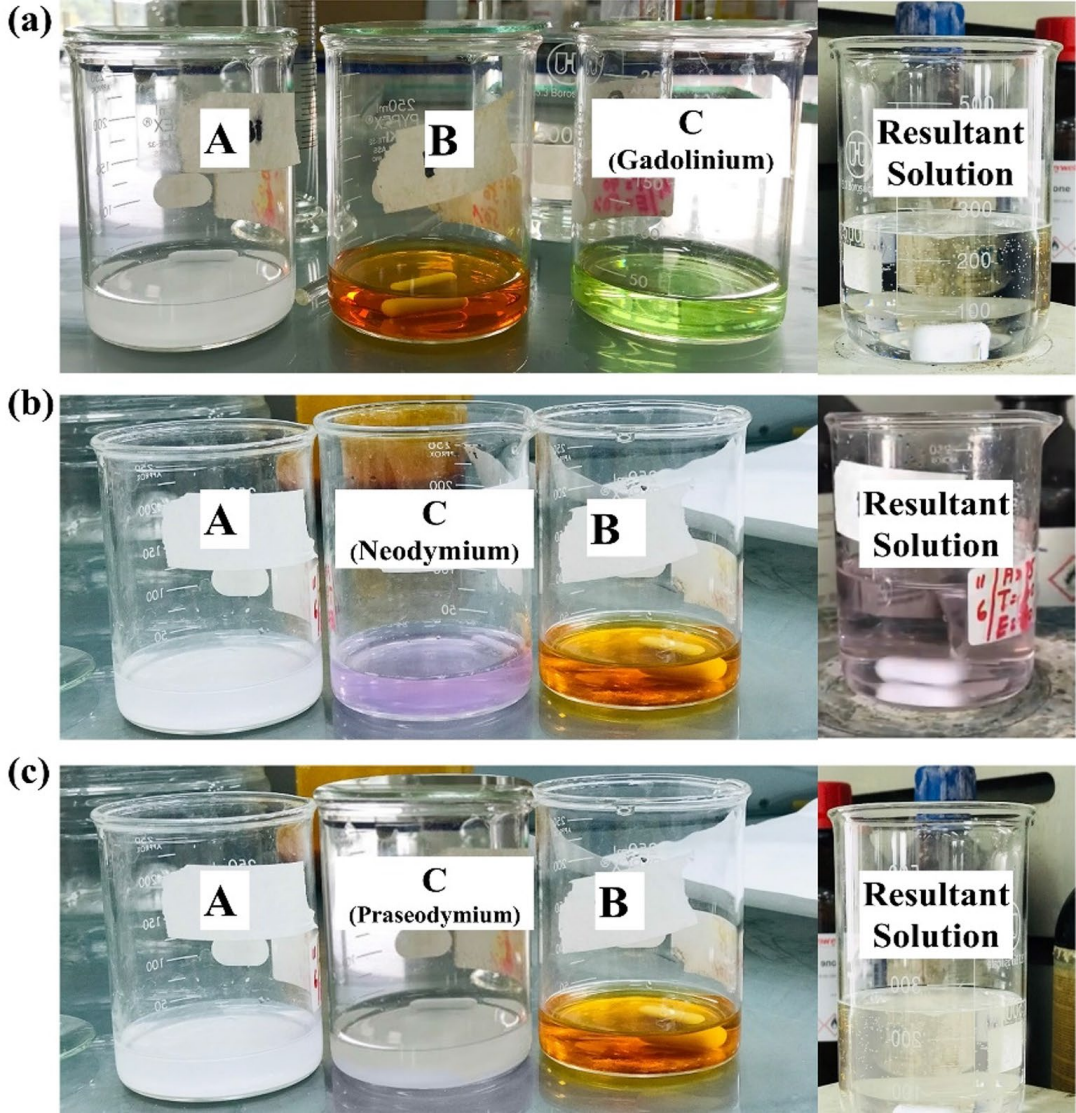
Doping of metal oxides with rare earth metals: Bi and Fe solutions with rare earth metals as (**a**) Gd solution and Gd doped BiFeO_3_, (**b**) Nd solution and Nd doped BiFeO_3_, and (**c**) Pr solution and Pr doped BiFeO_3_.

For the doping of BiFeO_3_ with rare-earth metals, 2.32 g of bismuth (III) nitrate pentahydrate (Bi(NO_3_)_3_·5H_2_O) was added to 35 mL of distilled water mixed with 15 mL of concentrated nitric acid and marked as solution “A”, whereas 3.61 g of iron (III) nitrate (Fe(NO_3_)_3_·9H_2_O) and 1.44 g of gadolinium (III) nitrate hexahydrate were dissolved and kept in two separate beakers and marked as solutions B and C, respectively (see Fig. 2a). Rare-earth metals, such as gadolinium (III) nitrate hexahydrate (Gd), neodymium (III) nitrate hexahydrate (Nd), and praseodymium (III) nitrate hexahydrate (Pr), with the amounts of 1.44 g, 1.52 g, and 1.54 g added into each beaker, respectively. For doping, the solutions B and C were mixed in a separate beaker, and then solution A was slowly added to the beaker containing solutions B and C, and the mixture containing all three solutions was hydrolysed to get precipitates, as shown in Fig. 2a–c. The obtained precipitates were calcined and ground according to the procedure stated above. A similar procedure was also adopted to synthesize Nd and Pr-doped BiFeO_3_ using 1.52 g and 1.54 g of neodymium (III) nitrate hexahydrate and praseodymium (III) nitrate hexahydrate, respectively, as can be seen from Fig. 2b and c.

### Fabrication of dye-sensitized solar cells

The DSSC devices have been fabricated by using different synthesized photoanode materials, and the activity of these cells has been evaluated by natural and artificial photosensitizers. All steps in this process are in order: cleaning the substrate (FTOs), preparing the working electrode (photoanode), preparing the dye solution (sensitizer), preparing the electrolyte, preparing the counter electrode (CE), and assembling the cell.

#### Cleaning of substrates

**Figure 3:**
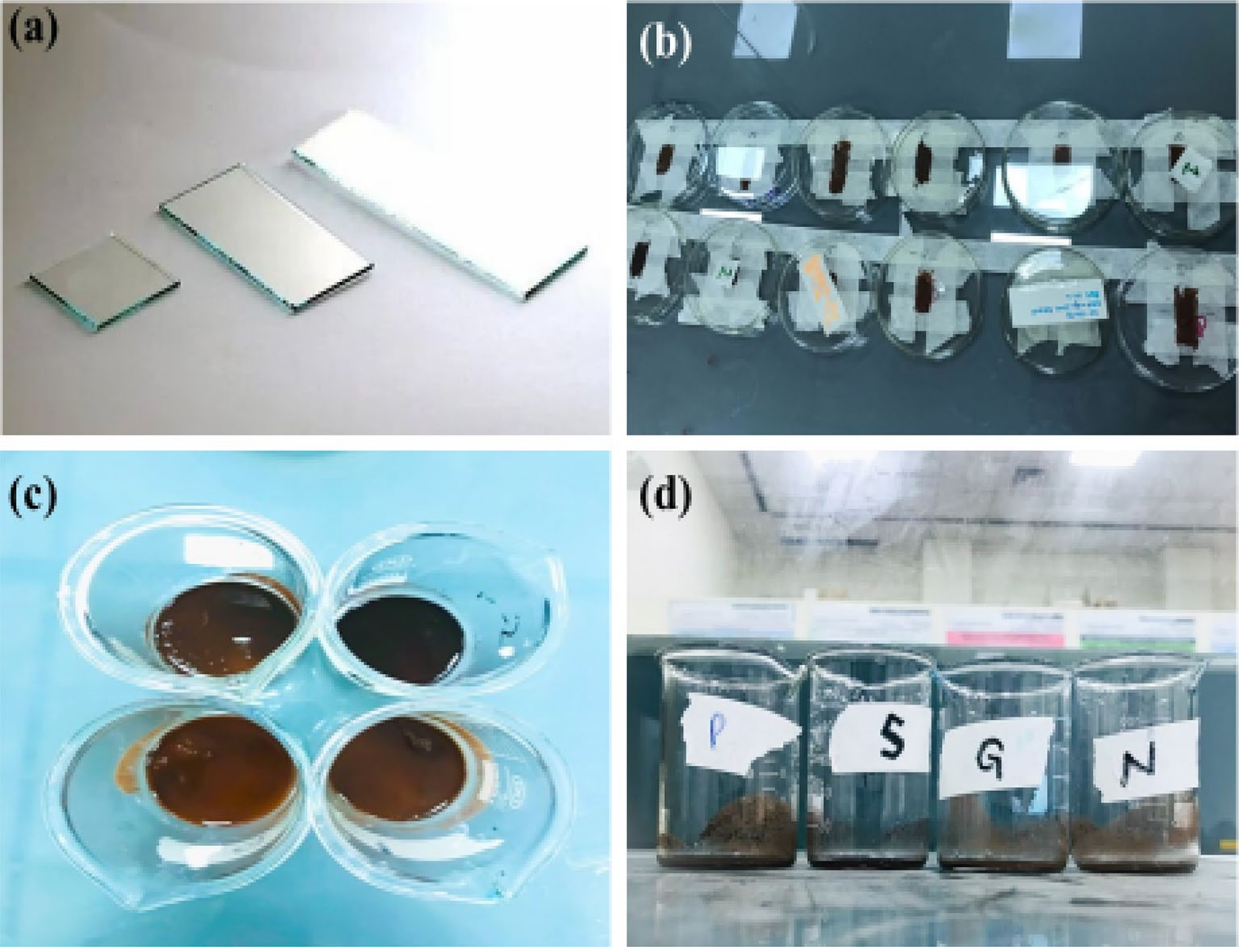
Preliminary setup for the preparation of photoanode: (**a**) FTO glass, (**b**) Doctor’s blade method, (**c**) Top view of the sonicated homogenized solution of bare/doped BiFeO_3_, and (**d**) Lateral view of sonicated homogenized solution of bare/doped BiFeO_3_.

Cleaning the substrate (conductive and transparent) is an important step in the preparation of DSSCs and enhancing their activity. Fluorine-doped tin oxide (FTO) glass was taken and cut into 2.5 × 1.5 cm rectangular pieces (see Fig. 3a). FTOs were cleaned in order to remove organic or inorganic impurities from the surface of the glass. The FTOs were then sonicated in acetone for 10 min, treated with ethanol and deionized water, and left to be dried for a while.

#### Preparation of working electrode (photoanode)

For the successful preparation of the photoanode, the Doctors' blade method^25^ was adopted as shown in Fig. 3b. The paste for photoanode material was prepared by weighing finely ground powder of 0.5 g. Then ethanol was taken (7 mL) and distilled water (3 mL) was added to it. After that, weighed BFO was mixed into an ethanol–water solution and gently mixed with the help of a glass rod. The solution was then subjected to a water bath sonicator and kept in the sonicator for 10 min until a thin and homogenous paste was obtained (see Fig. 3c, d). For the preparation of the working electrode (WE), the conductive surface of FTO glass was confirmed by using a multimeter and it covers its whole surface (1 cm × 1 cm). A paste of photoanode material was applied on the (uncovered) conductive side of the FTO substrate and left to dry for 24 h. After the film dried at room temperature, the tape was carefully removed and placed into the furnace at a temperature of 100 °C for 4 h. The same procedure was repeated to prepare the paste for all modified materials.

#### Preparation of dye solution (sensitizer)

**Figure 4:**
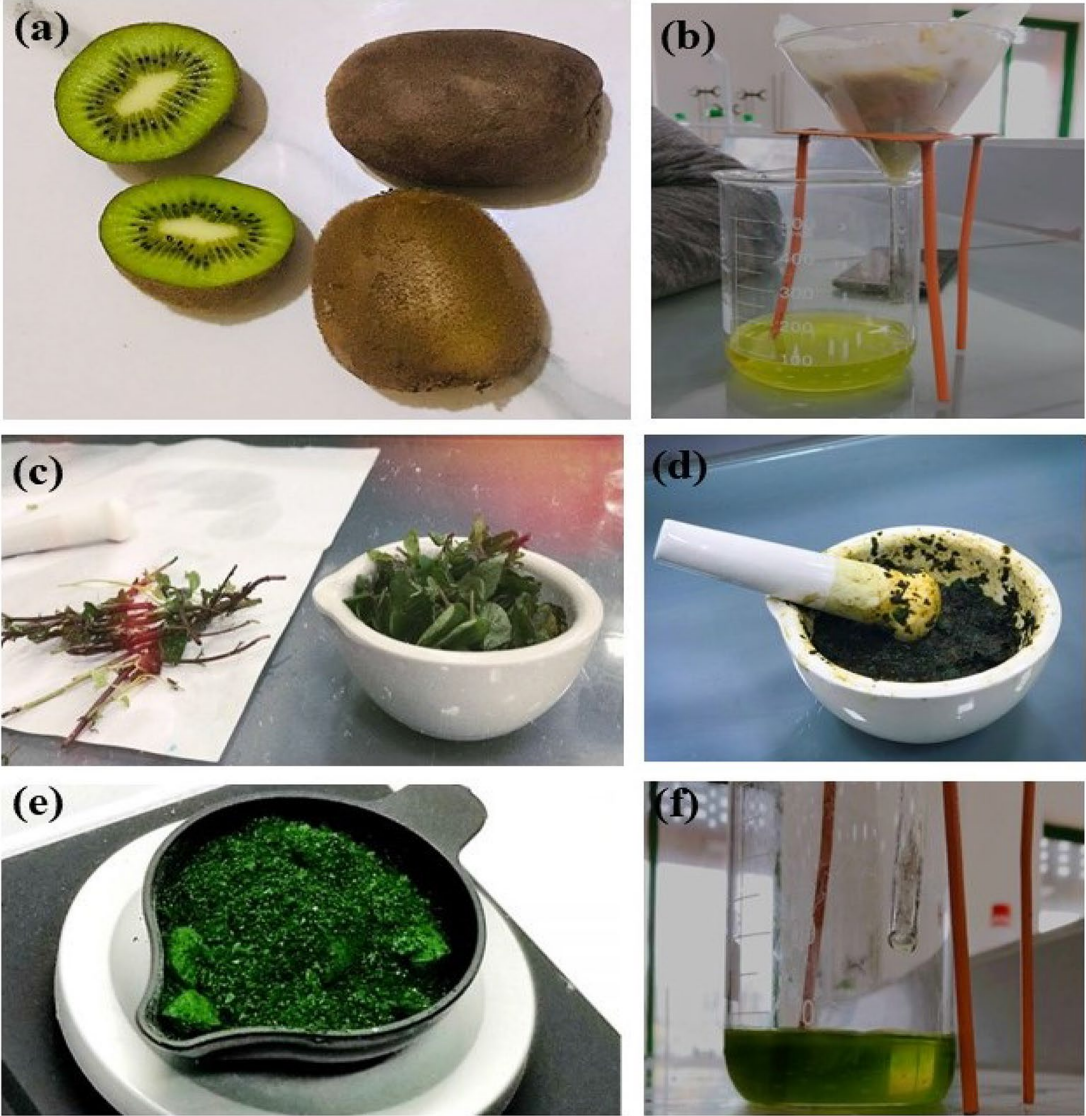
Preliminary setup for the preparing sensitizer: (**a**) Kiwi (*Actinidia deliciosa*), (**b**) Kiwi (*Actinidia deliciosa*) extract, (**c**) Mint leaves (*Mentha*), (**d**) Extraction of mint leaves (*Mentha*), (**e**) Synthetic dye malachite green, and (**f**) Extraction of synthetic dye malachite green.

The preparation of the sensitizer involves two steps: the extraction of a natural dye and the preparation of a synthetic dye solution. For the extraction of natural dye, kiwi (*Actinidia deliciosa*) (see Fig. 4a) and mint leaves (*Mentha*) (see Fig. 4c), which were obtained from the local market supplier in Lahore, Pakistan, were washed thoroughly, then ground them gently with a mortar and pestle while adding 50 mL of ethanol. After the successful grinding process, the extracted dye was filtered as shown in Fig. 4b and d. The residues were discarded, and the filtrate was collected in a culture bottle and covered with aluminium foil for further use. For the preparation of synthetic dye, malachite green was taken (see Fig. 4e), and a 10-ppm solution was prepared in a 100-mL conical flask. For this purpose, 1 mg of malachite green was taken in the flask along with distilled water. The solution was shaken vigorously until the dye was completely dissolved. After the complete dissolution of the dye, distilled water was carefully added up to the mark of 100 mL (see Fig. 4f).

#### Preparation of electrolyte

Iodide tri-iodide (I^−^/I^−^_3_) was used as an electrolyte to fill the gap between the electrodes. To make the electrolyte, 10 mL of ethylene glycol was placed in 50 mL electrodes. For the preparation of the electrolyte, 10 mL of ethylene glycol was put in a 50-mL beaker, and then 0.127 g of iodine was added to the beaker. Then 0.83 g of potassium iodide was added to the beaker. The components of the beaker were stirred and slightly heated by using a hot plate until a clear solution was obtained. When the solution was formed, the beaker was completely covered with aluminium foil and stored in a dark place.

#### Preparation of counter electrode

**Figure 5:**
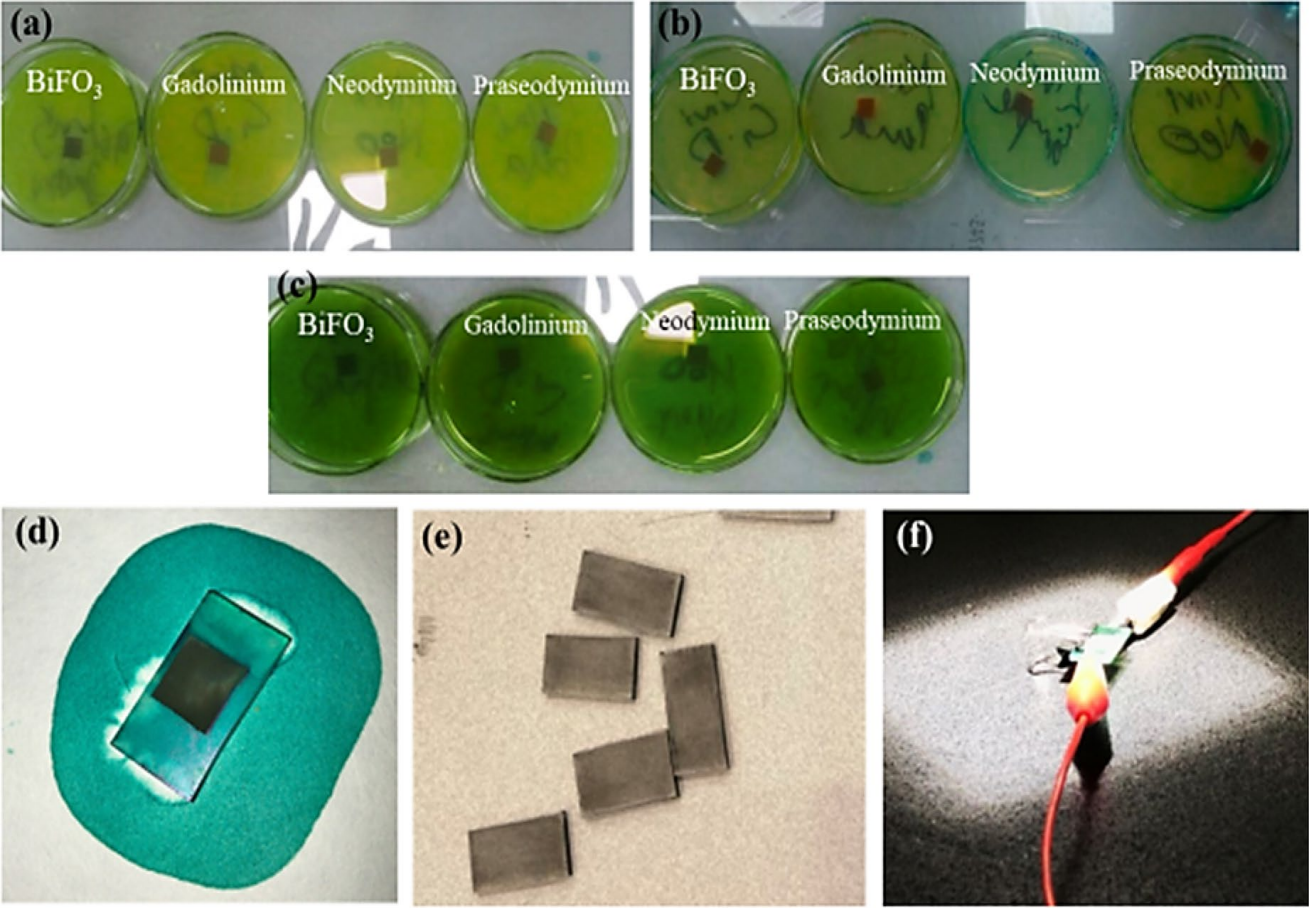
Method of assembling of the cell: (**a**) Photoanodes immersed in mint dye (*Mentha*), (**b**) Photoanodes immersed in Kiwi dye (*Actinidia deliciosa*), (**c**) Photoanodes immersed in the synthetic malachite green dye, (**d**) Photoanode taken out from the dye, (**e**) Carbon coated FTOs, and (**f**) Assembly of a DSSC.

The preparation of the counter electrode (CE) for the fabrication of dye-sensitized solar cells was done by identifying the conductive surface of washed FTOs via ammeter and coating the conductive surface of FTOs with the help of a lead pencil in order to apply a carbon layer on this surface (see Fig. 5e). The pencil was rubbed on FTO in a criss-cross pattern so that no spot was left behind.

#### Assembling the cell

After the preparation of all the components, the cell was assembled and prepared for testing. In the first step, the prepared photoanode was dipped in the solution of both the natural and synthetic dyes for 24 h (see Fig. 5a–c), then the FTOs (photoanodes) were gently removed from the solution, washed with distilled water, and left to dry (see Fig. 5d). After that, 1–2 drops of electrolyte were introduced to the photoanode with the help of a dropper. In the end, the counter electrode was placed over the working electrode and sealed with the help of binding clips (see Fig. 5f). Then the cell was subjected to the solar simulator to obtain the I–V curves. The same procedure was repeated to prepare DSSCs using all the modified nanoparticles.

For the evaluation of the power conversion efficiency of the fabricated solar cells, their activity was measured using a solar simulator, the SS50AAA. Moreover, the following mathematical expressions have been used for the determination of fill factor (FF) and power conversion efficiency^26^:

1 $$FF=\frac{{I}_{mP}\times {V}_{{m}_{P}}}{{I}_{sc}\times {V}_{oc}}$$

The following relationship has been used for the calculation of the power conversion efficiency, $\eta [{\%}]$:

2 $$\eta =\frac{F\cdot F\times {I}_{sc}\times {V}_{oc}}{\left(\frac{\mathrm{mW}}{{\mathrm{cm}}^{2}}\right)\times \left(Area\right)}[\%]$$

## Research involving plants statement

The use of plant/plant parts in the present study complies with international, national, and/or institutional guidelines.

## Results and discussion

The synthesized BiFeO_3_ and rare-earth metal doped BiFeO_3_ materials were characterized through different techniques for their optical (UV-DRS and PL), structural (XRD), and morphological (SEM) evaluation.

### UV–visible diffuse reflectance spectroscopy (UV–vis DRS)

**Figure 6:**
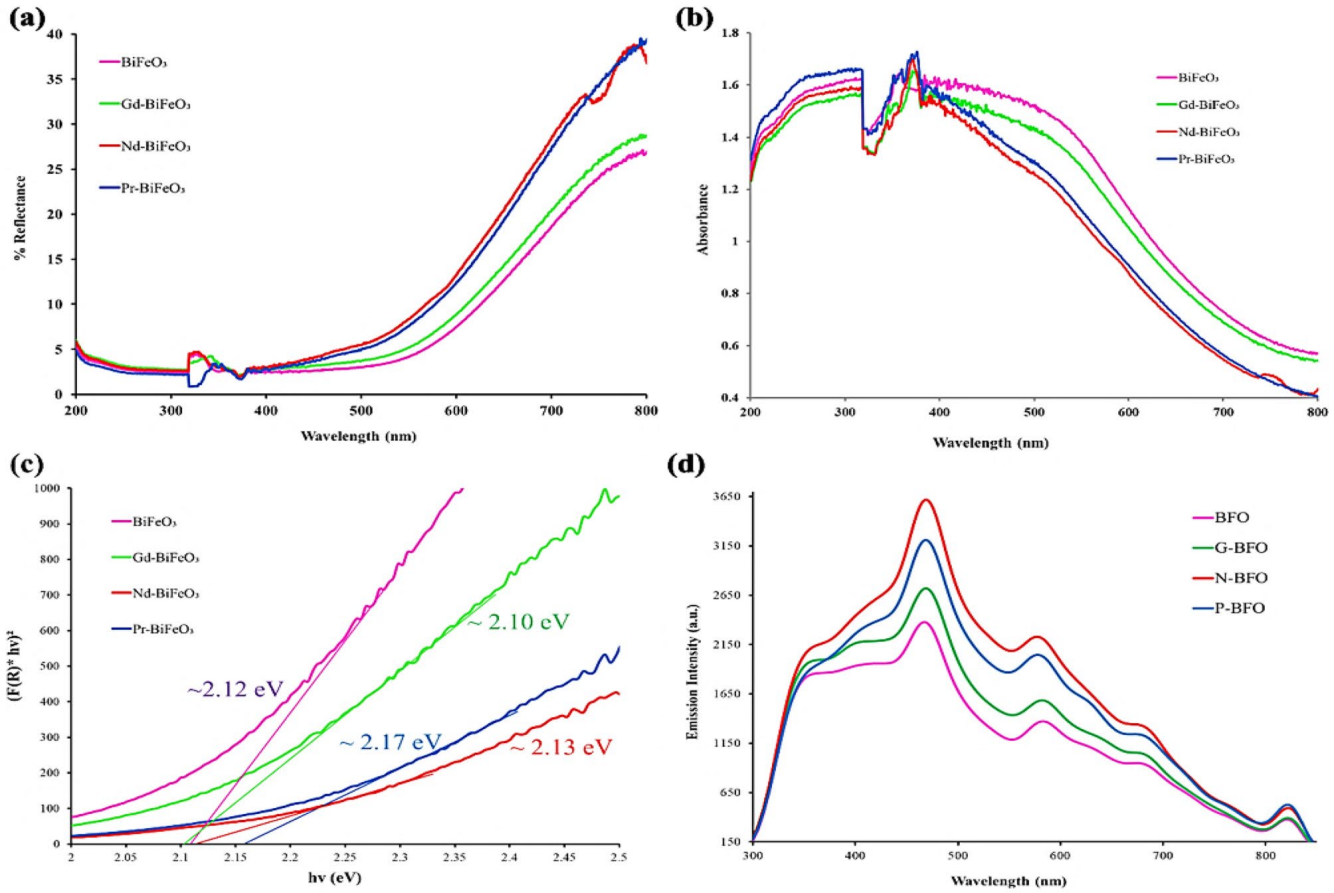
(**a**) UV–Vis diffuse reflectance spectra of synthesized bare/doped BiFeO_3_, (**b**) UV–Vis diffuse absorbance spectra of synthesized bare/doped BiFeO_3_, (**c**) Tauc plots against[F(R)*hv]^2^ versus hv (eV), and (**d**) Photoluminescence spectra of synthesized bare/doped BiFeO_3_.

A diffuse reflectance spectrophotometer (DRS) works on the principle of reflected light coming out of the surface of a solid material. The UV-DRS was used to measure the optical reflectance as well as the absorbance of the synthesized materials. Reflectance spectra indicated that strong reflectance peaks have been observed around 500–600 nm, which means that material has a strong percentage reflectance in the visible region as depicted in Fig. 6, wherein high reflectance peaks were shown by Nd-doped BiFeO_3_ and Pr-doped BiFeO_3_, while low reflectance peaks were shown by Gd-doped BiFeO_3_ and bare BiFeO_3_, respectively (see Fig. 6a). The absorption spectra of pristine BiFeO_3_ have shown the highest absorption in the visible region. As the material is doped with rare-earth metals, there is lower absorption observed in the visible region, as shown in Fig. 6b. With the addition of Gd and Nd, a slightly lower absorption spectrum has been observed than that of simple BFO. Meanwhile, the spectra of Pr-doped BiFeO_3_ material presented the lowest absorption in the visible region, while in the UV-ultraviolet region, a different response has been observed. All the materials have shown remarkable absorbance in the UV-ultraviolet region. Peak shifting can be clearly seen in the absorption patterns of the materials, from highest to lowest for Pr-doped BiFeO_3_, Nd-doped BiFeO_3_, BiFeO_3,_ and Gd-doped BiFeO_3_. It has also been observed that there is no additional notable peak appeared in the spectra of doped material, which shows a good agreement of rare-earth metal in the host material. As the absorption is related to excitation and is dependent on the electron population, the UV or VIS response regions for all materials may vary significantly^27^.

Moreover, the Tauc plot given in Fig. 6c shows the bandgap of each synthesized material and concludes that the bandgap of bare BiFeO_3_ is ~ 2.12 eV, while the bandgap of doped nanoparticles such as Gd-doped BiFeO_3_, Nd-doped BiFeO_3_ and Pr-doped BiFeO_3_ is found to be ~ 2.10, ~ 2.13 and ~ 2.17 eV, respectively. A slight shift in bandgap has been observed for all the materials, such as the bandgap of bare BiFeO_3_ being lower than that of Gd-doped BiFeO_3_, while it is nearly equal to the bandgap of Nd-doped BiFeO_3_. On the other hand, Pr-doped BiFeO_3_ has a higher bandgap energy than that of bare BiFeO_3_. Hence, there is no significant change in the bandgap. So, it can be concluded that if the number of rare-earth metals (doping material) varies, then a recognizable change in the bandgap can be seen. Moreover, there is a slight difference between the bandgap of bare and doped BiFeO_3,_ and the PL emission intensity of bare BiFeO_3_ is observed to be lower than that of doped material (see Fig. 6d) because the sub-energy bands formed are very close to each other below the conduction band, which ultimately leads to the minor energy difference.

### Photoluminescence spectra analysis

The optical properties of pristine BiFeO_3_ and doped samples, such as Gd-doped BiFeO_3_, Nd-doped BiFeO_3,_ and Pr-doped BiFeO_3_ have been investigated from the analysis of photoluminescence (PL) emission spectra. The emission peaks are seen at around 490 nm for all the samples, as shown in Fig. 6d. Emission spectra can be used to analyze the concentration of emitted electrons at a specific wavelength, from which it has been observed that doped materials have lower emission intensities as compared to simple BFO. However, the short lifespan of excited electrons in doped materials in the conduction band is due to the gradual concentration-quenching effect. Another reason for increasing emission intensity is the creation of doping trap states that are very close to each other and have no energy difference, which gives rise to the emission of electrons^28^.

### Morphological analysis

**Figure 7:**
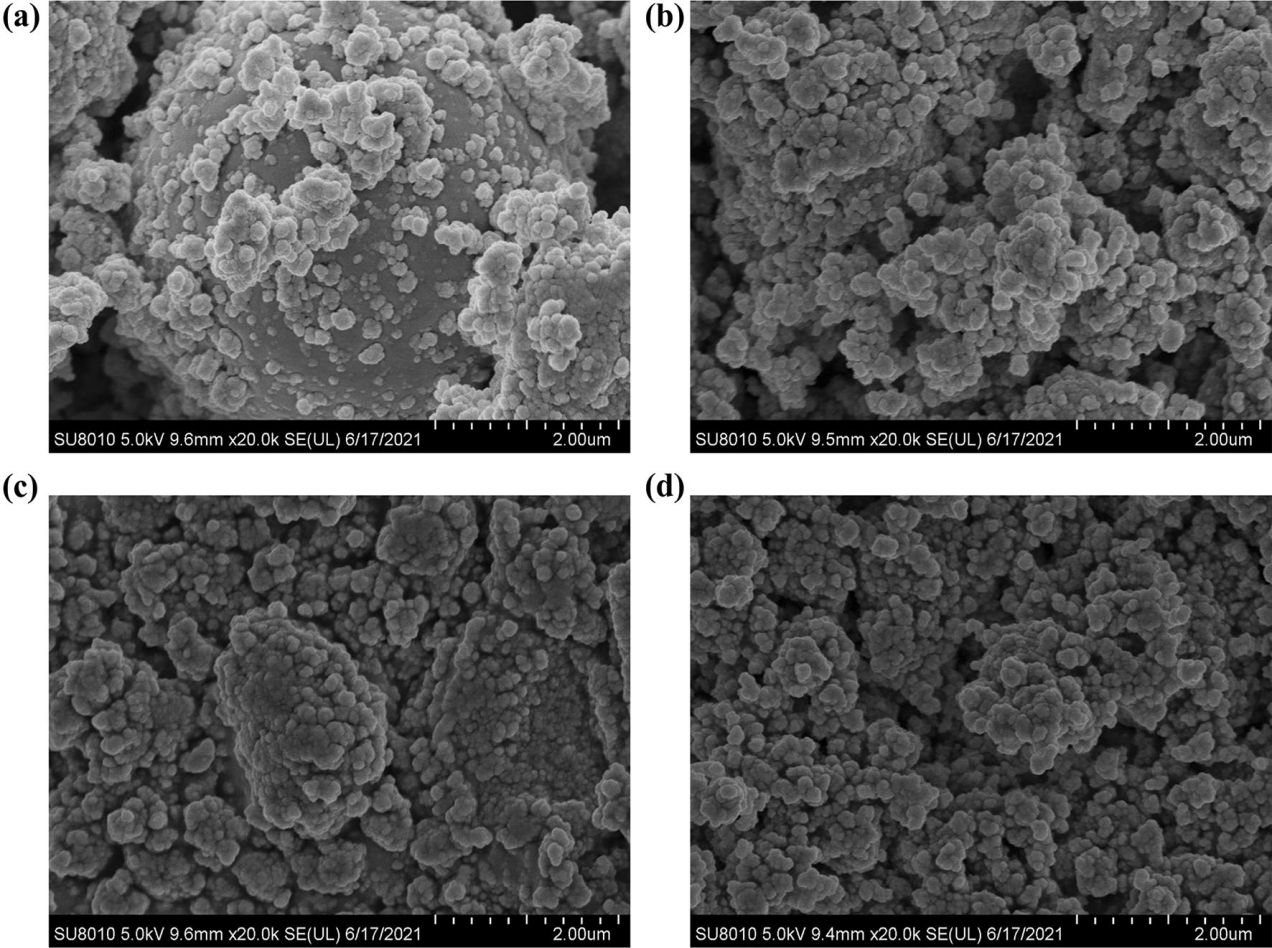
SEM micrographs of the synthesized materials: (**a**) BiFeO_3_, (**b**) Gd-BiFeO_3_, (**c**) Nd-BiFeO_3_, and (**d**) Pr-BiFeO_3_.

A scanning electron microscope (SEM) analysis was performed, and this provided us with an idea of the grain size. Figure 7 shows the SEM micrographs of the prepared samples.

**Figure 8:**
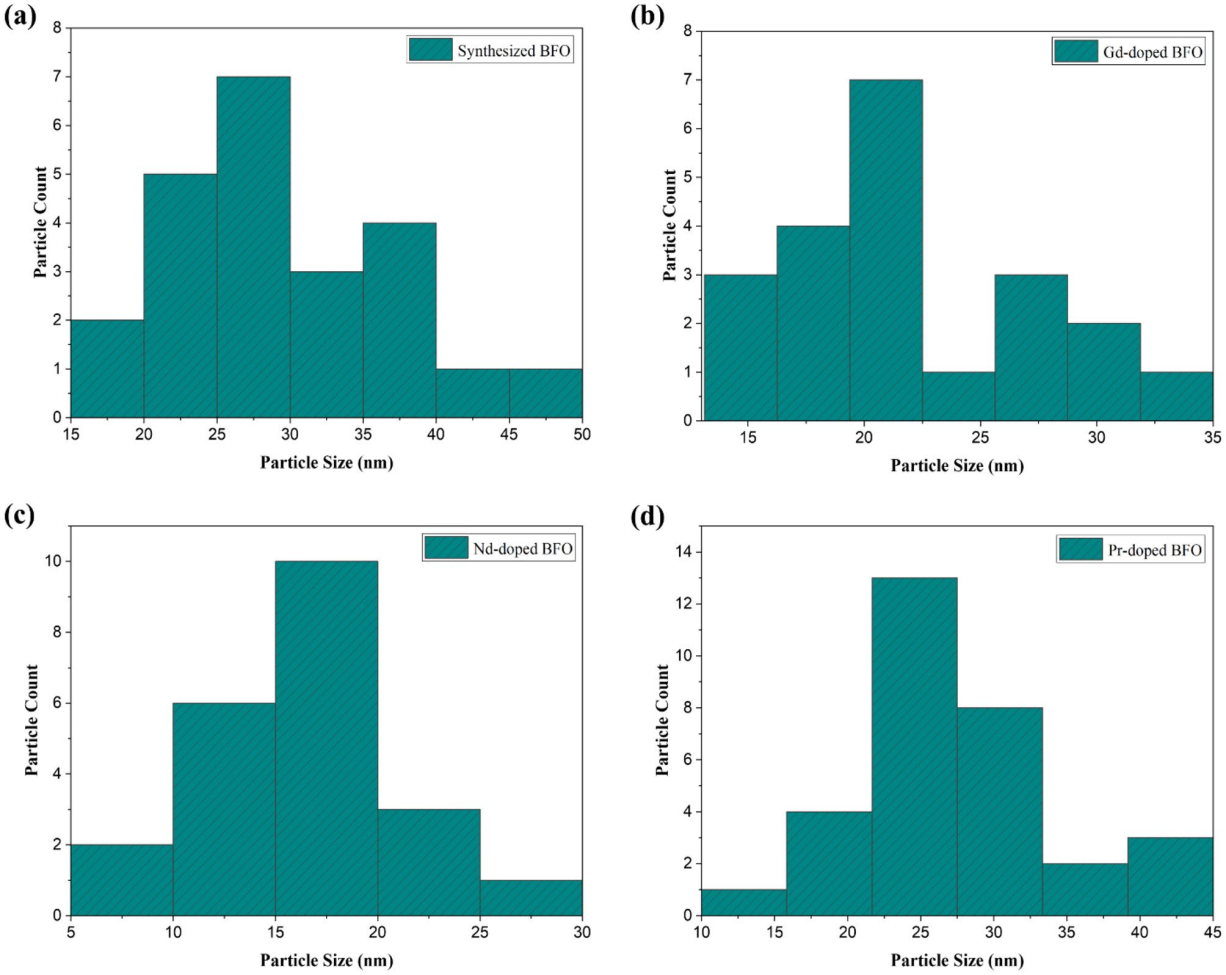
Particle size distribution of: (**a**) Synthesized BFO, (**b**) Gd-doped BFO, (**c**) Nd-doped BFO, and (**d**) Pr-doped BFO.

The grain size was well-developed in this experiment where it is non-uniform, wherein BFO was observed with the largest particle size, estimated to be around 25–30 nm with the maximum number of particles (see Fig. 8a), while it has been observed that the particle size appeared to be decreasing in the case of rare earth doping (see Fig. 7a). The average size of observed Gd-dopped BFO particle sizes was about 20 nm (see Figs. 7b and 8b), while Nd-dopped and Pr-dopped BFO nanoparticle sizes were calculated and found to be around 17 nm and 25 nm, respectively (see Figs. 7, and 8c, d). During the doping process, the particles agglomerate while the particle size starts to decrease. However, the morphology of particles remains the same as spherical.

Figure 8 depicts the particle size distribution of the synthesized bare and rare earth doped BFO nanoparticles, with the bare BFO particles being the largest, measuring 15–50 nm. However, the Nd-doped BFO particles were the smallest of their counterparts, in the range of 5–30 nm. The particle sizes of Gd-doped and Pr-doped particles were 14–35 nm and 10–45 nm, respectively. This particle size distribution was calculated by using the ImageJ (Version 1.53 m) software^29^.

### X-ray diffraction analysis

**Figure 9:**
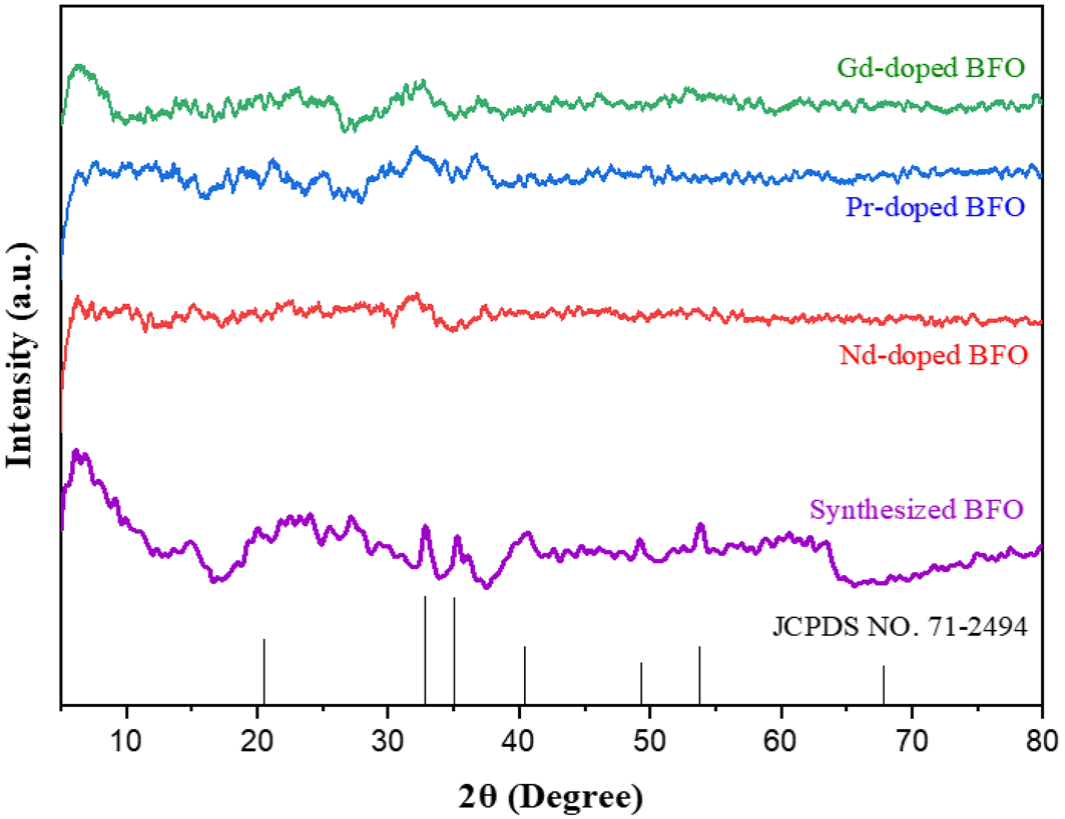
XRD pattern analysis of synthesized bare as well as Nd, Pr, and Gd-doped BiFeO_3_ materials.

The X-ray diffraction (XRD) patterns for bare BiFeO_3_ and doped materials such as Gd-doped BiFeO_3_, Nd-doped BiFeO_3_, and Pr-doped BiFeO_3_ are displayed in Fig. 9. The XRD analysis shows the crystalline nature of the synthesized BFO in comparison to the reference BFO XRD graph from JCPDS No. 71-2494^30^, appearing sharp peaks at 2θ values at 20.5°, 32.1°, 35.3°, 41.6°, 49.4°, and 67.7°. However, for the rare earth metal doped with BFO, the height of the peaks exhibits the low crystallinity and amorphous background of the material, along with the impurity phases and residual amorphous precursors. When rare earth metals were added to the BiFeO_3_ host material, complete structural and phase transformations were observed. Peak splitting can be clearly observed from the XRD spectra of doped material when comparing it with the reference BFO material, and this exhibits the pure crystalline nature of the reference BFO material. Peak intensity is directly proportional to the number of scatters per unit area. Peak intensity is directly proportional to the number of scatters per unit area. However, too many peaks in the XRD analysis demonstrate the existence of noise, which indicates the low crystallinity and amorphous nature of the materials. No prominent peaks are observed in this case, but only the small peaks associated with clear distortions^31^.

### Evaluation of photovoltaic parameters of DSSCs

**Figure 10:**
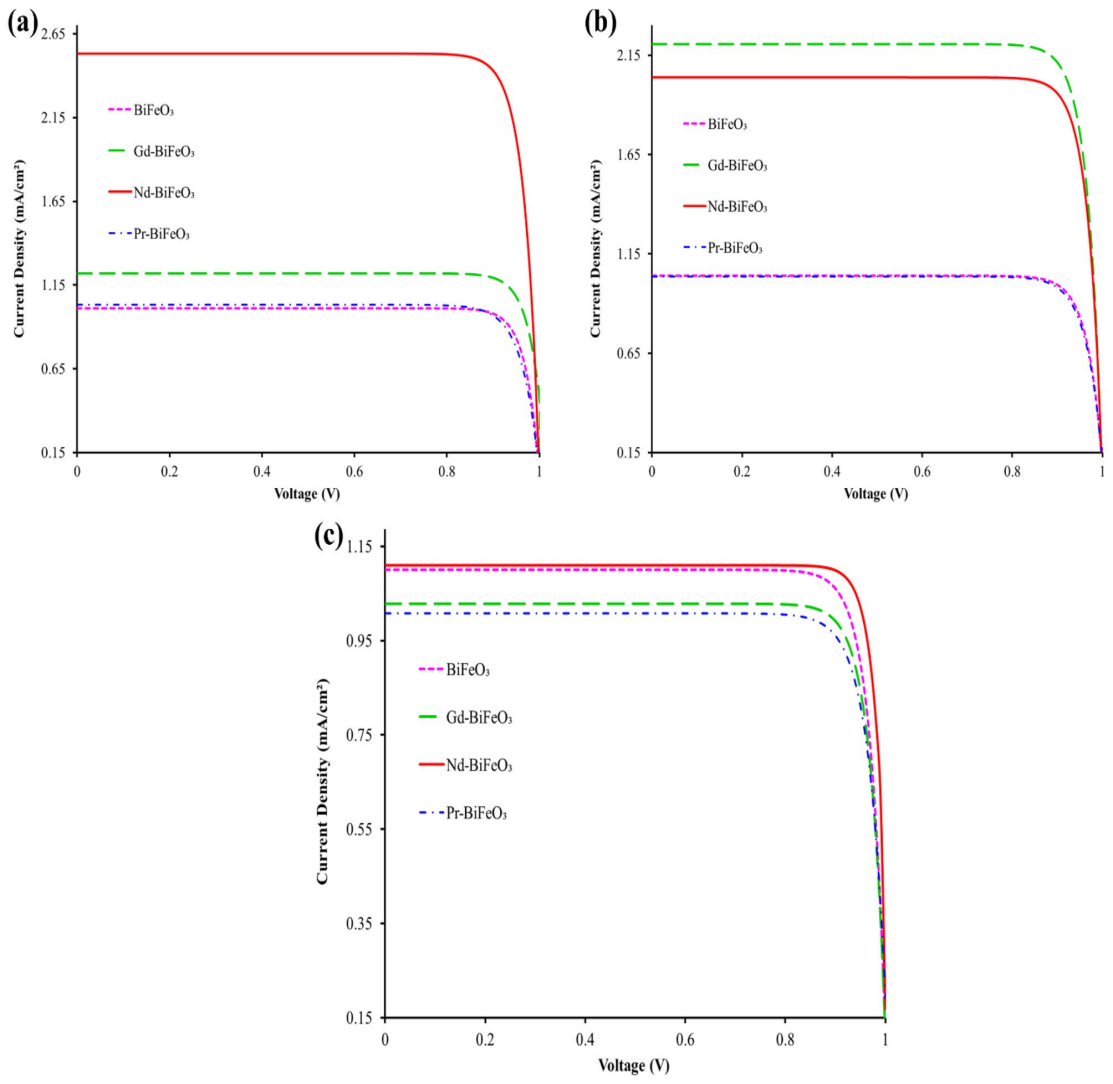
I–V curves of DSSC based on photoanodes by using: (**a**) Mint (*Mentha*), (**b**) Kiwi (*Actinidia deliciosa*), and (**c**) Malachite green dye solution.

Evaluation of various parameters of fabricated dye-sensitized solar cells based on synthesized materials using extracted mint *(Mentha),* kiwi (*Actinidia deliciosa),* and malachite green dye (sensitizer) were studied, together with the testing of I–V curves of twelve fabricated DSSCs (see Fig. 10). Tables 1, 2 and 3 present different photovoltaic parameters like short circuit current density (J_sc_) and open circuit voltage (V_oc_), fill factor (FF), area (cm^2^), and efficiency (η%). Interestingly, three different sensitizers have been employed to investigate the photovoltaic parameters. The value of short-circuit current density (J_sc_) using *Mentha, Actinidia deliciosa,* and malachite green sensitizers was recorded as ranging from 1.01% to 2.53%, 1.03% to 2.20, and 0.99% to 1.11%, respectively, as shown in Fig. 10a–c. The corresponding value of fill factor (FF) lies between 0.82 and 0.89%, 0.84–0.86%, and 0.85–0.88%, respectively. The power conversion efficiencies (η%) of the DSSCs are recorded as ranging from 0.85% to 2.15%, 0.86% to 1.87%, and 0.84% to 0.98%, respectively.

**Table 1:** Photovoltaic parameters of the DSSC fabricated by using an extracted mint (*Mentha*) dye solution.

Device	J_sc_ (mA cm^−2^)	V_oc_ (V)	FF	Area (cm^2^)	*ƞ* (%)
BiFeO_3_	1.01	1.00	0.89	1	0.89
Gd doped BiFeO_3_	1.22	1.00	0.85	1	1.04
Nd doped BiFeO_3_	2.53	1.00	0.85	1	2.15
Pr doped BiFeO_3_	1.03	1.00	0.82	1	0.85

**Table 2:** Photovoltaic parameters of the DSSC fabricated by using an extracted Kiwi (*Actinidia deliciosa*) dye solution.

Device	J_sc_ (mA cm^−2^)	V_oc_ (V)	FF	Area (cm^2^)	*ƞ* (%)
BiFeO_3_	1.04	1.00	0.85	1	0.90
Gd doped BiFeO_3_	2.20	1.00	0.85	1	1.87
Nd doped BiFeO_3_	2.04	1.00	0.86	1	1.76
Pr doped BiFeO_3_	1.03	1.00	0.84	1	0.86

**Table 3:** Photovoltaic parameters of the DSSC fabricated by using malachite green dye solution.

Device	J_sc_ (mA cm^−2^)	V_oc_ (V)	FF	Area (cm^2^)	*ƞ* (%)
BiFeO_3_	1.09	1.00	0.86	1	0.94
Gd doped BiFeO_3_	1.02	1.00	0.85	1	0.87
Nd doped BiFeO_3_	1.11	1.00	0.88	1	0.98
Pr doped BiFeO_3_	0.99	1.00	0.85	1	0.84

It has been observed that the PCE of bare BiFeO_3_ is lower as compared to the doped photoanodes, such as Gd-doped BiFeO_3_, Nd-doped BiFeO_3_, and Pr-doped BiFeO_3_ but it is still not high enough. The low output PCE is due to the fact that the electron transfer mechanism of dye-sensitized solar cells is limited due to the trap states that occur after doping, which ultimately affects the recombination process of electron movement in DSSCs. These trap states can sometimes enhance the efficiency of solar cells, and sometimes they can cause losses in devices^32^. Although, in doped photoanodes, increasing the amount of dopant can increase the number of these trap states, causing recombination and lowering solar cell efficiency. Furthermore, in doped photoanodes, there is a high refractive index that gives rise to effective diffused scattering of light inside the porous thin layer of the photoanode, which considerably increases light absorption. A decrease in energy gap shifts the optical absorption to a longer wavelength because of the receipt of a large number of photons in doped photoanodes. The enhancement of the light harvesting yield of dye-sensitized solar cells can also enhance the short-circuit photocurrent (J_sc_) in dye-sensitized solar cells, which ultimately increases the PCE of the DSSCs^33^. The PCE of fabricated DSSC was calculated, and the use of doped material improves the dye loading capacity of the BFO nanoparticles, demonstrating the high efficiency of Mentha dye-involving DSSCs originated from their efficient functional groups’ involvement. Electron trapping behavior enhances the number of photo-generated charge carriers, which ultimately leads to low recombination of charges and hence high PCE in DSSCs^34^.

The photovoltaic parameters of fabricated DSSCs derived from rare-earth metals modified BiFeO_3_ have been computed in relation with the use of different sensitizers. The results of that computation are summarized in tables, where Table 1 is for the extracted mint dye solution (Mentha), Table 2 for the extracted Kiwi dye solution (Actinidia deliciosa), and Table 3 for the extracted Malachite Green dye solution. Both V_oc_ and FF are also important parameters in a solar cell that depends upon the temperature, initial current density, saturation current, light-generated current, and thickness of an absorber, along with the size of nanoparticles. V_oc_ is also associated with the forward-biased current that is generated by photoelectrons, and it increases when the temperature grows. Whereas FF is a measure of the solar cell quality, which is linked to the thickness of the absorbing layer, the increase in layer thickness leads to absorbing low intensity irradiation and hence to increase of the value of FF^35^.

### Comparison between PCE of DSSCs

A comparison between twelve fabricated DSSCs was analyzed, where three different dyes or sensitizers were used, such as mint (*Mentha*), kiwi (*Actinidia deliciosa*), and malachite green, to fabricate the DSSCs, and their corresponding *ƞ* (%) was recorded. The recorded efficiency shows that rare earth-doped BFO has a higher PCE than pure BFO, while the dyes also play a vital role in improving the PCE. Sensitizers, photoanodes, electrolytes, and conducting materials play parallel roles in the fabrication of efficient DSSCs. Due to the less stable and less long-lasting nature of natural dyes, calculated efficiencies of them are found to be low. The use of a suitable dye as a sensitizer along this line plays a major role in improving performance of DSSC. An efficient dye can be identified by its absorption capacity of photons in the visible light spectrum or near the IR spectrum. However, the photoanode acts as a supporter to load the sensitizer and to transport photoelectrons from the sensitizer to the external circuit^36,37^. According to the findings of this study, mint dye is proven to be a good sensitizer, with Nd-doped BFO achieving a maximum efficiency of 2.15%, followed by rare earth-doped BFO in the kiwi sensitizer achieving an efficiency range of 1.7–1.9%. Natural sensitizers are eco-friendly and cost-effective in addition to their high PCE properties, while synthetic dye-based materials have very low PCE compared to their counterparts.

## Conclusions

In this research, BiFeO_3_ was synthesized and successfully modified with rare-earth metals such as Nd, Gd, and Pr by employing the co-precipitation method. UV–Vis DRS investigated reflectance spectra that depicted the strong reflectance and absorption peaks in the visible region, which, with the addition of Gd and Nd, observed a slightly lower absorption spectrum than the simple BFO. In addition, the detailed morphology was confirmed by a SEM, confirming the well-developed spherical particle size in the range of 5–50 nm, while XRD spectra presented the phase transition from crystalline to amorphous, wherein they showed crystalline behavior for bare BFO while showing the amorphous nature of doped materials. The PCE of fabricated DSSC was calculated, and it has been observed that doped material resulted in improving the dye loading capacity of the BFO nanoparticles, demonstrating the high efficiency of *Mentha* dye-involving DSSCs owing to their efficient functional groups involvement, and the PCE ranges from 0.84 to 2.15%, confirming that mint (*Methna*) and Nd-doped BiFeO_3_ (the smallest size nanoparticles of their counterparts, in the range of 5–30 nm) were found to be the most efficient sensitizer and photoanode, respectively. Sensitizers and photoanodes used in this research are also eco-friendly and cost-effective by nature.

## Data Availability

All the data generated or analyzed during this study has been included in this published article.

## References

[1] Lotey GS, Verma NK (2013). Gd-doped BiFeO_3_ nanoparticles—A novel material for highly efficient dye-sensitized solar cells. Chem. Phys. Lett..

[2] Lotey GS, Verma NK (2014). Synthesis and characterization of BiFeO_3_ nanowires and their applications in dye-sensitized solar cells. Mater. Sci. Semicond. Process..

[3] Verma NK, Kaur I, Kaur K, Lotey GS (2014). Enhanced efficiency of Au-deposited BiFeO_3_ nanoparticles based dye-sensitized solar cells. Adv. Mater. Res..

[4] Aslam A, Mehmood U, Arshad MH, Ishfaq A, Zaheer J, Khan AUH, Sufyan M (2020). Dye-sensitized solar cells (DSSCs) as a potential photovoltaic technology for the self-powered internet of things (IoTs) applications. Sol. Energy.

[5] Kumar TN, Yuvaraj S, Kavitha P, Sudhakar V, Krishnamoorthy K, Neppolian B (2020). Aromatic amine passivated TiO_2_ for dye-sensitized solar cells (DSSC) with ~ 9.8% efficiency. Sol. Energy..

[6] Omar A, Ali MS, Abd Rahim N (2020). Electron transport properties analysis of titanium dioxide dye-sensitized solar cells (TiO_2_-DSSCs) based natural dyes using electrochemical impedance spectroscopy concept: A review. Sol. Energy.

[7] Ghernaout D, Boudjemline A, Elboughdiri N (2020). Charge neutralization in the core of plasma treatment. OALib.

[8] Mohamed MM, Reda SM, Amer AA (2020). Enhanced performance of BiFeO_3_@ nitrogen doped TiO_2_ core-shell structured nanocomposites: Synergistic effect towards solar cell amplification. Arab. J. Chem..

[9] Sahni M, Kumar D, Chauhan S, Singh M, Kumar N (2020). Study of structural, optical and photocatalytic activity of Sm and Ni doped BiFeO_3_ (BFO) and BFO@ ZnO nanostructure. Mater. Today Proc..

[10] Yu L, Wang L, Dou Y, Zhang Y, Li P, Li J, Wei W (2022). Recent advances in ferroelectric materials-based photoelectrochemical reaction. Nanomaterials.

[11] Kadi MW, Mohamed RM, Ismail AA (2020). Facile synthesis of mesoporous BiFeO_3_/graphene nanocomposites as highly photoactive under visible light. Opt. Mater..

[12] Li S, Zhang G, Zheng H, Zheng Y, Wang P (2017). Stability of BiFeO_3_ nanoparticles via microwave-assisted hydrothermal synthesis in Fenton-like process. Environ. Sci. Pollut. Res. Int..

[13] Li S, Lin Y-H, Zhang B-P, Wang Y, Nan C-W (2010). Controlled fabrication of BiFeO_3_ uniform microcrystals and their magnetic and photocatalytic behaviors. J. Phys. Chem. C.

[14] Wang B, Biesold GM, Zhang M, Lin Z (2021). Amorphous inorganic semiconductors for the development of solar cell, photoelectrocatalytic and photocatalytic applications. Chem. Soc. Rev..

[15] De AK, Majumdar S, Pal S, Kumar S, Sinha I (2020). Zn doping induced band gap widening of Ag_2_O nanoparticles. J. Alloys Compd..

[16] Hou J, Zhou J, Liu Y, Yang Y, Zheng S, Wang Q (2020). Constructing Ag_2_O nanoparticle modified TiO_2_ nanotube arrays for enhanced photocatalytic performances. J. Alloys Compd..

[17] An’Amt MN, Radiman S, Huang NM, Yarmo MA, Ariyanto NP, Lim HN, Muhamad MR (2010). Sol–gel hydrothermal synthesis of bismuth–TiO_2_ nanocubes for dye-sensitized solar cell. Ceram. Int..

[18] Karki IB, Nakarmi JJ, Mandal PK, Chatterjee S (2013). Absorption spectra of natural dyes and their effect on efficiency of ZnO based dye-sensitized solar cells. NJST.

[19] Kim JH, Kim DH, So JH, Koo HJ (2021). Toward eco-friendly dye-sensitized solar cells (DSSCs): Natural dyes and aqueous electrolytes. Energies.

[20] Lachore WL (2022). Zinc oxide nonmaterial based dye-sensitized solar cells using natural dyes extracted from different plant pigment. Am. J. Mod. Energy.

[21] Nirmala M, Sahana S, Iswarya B, Maruvarasi K, Jenita A, Kavitha B (2020). Fabrication of dye sensitized solar cell based on natural photosensitizers. World Sci. News.

[22] Prabu KM, Anbarasan PM (2015). Preparation and performance study of dye sensitized solar cells using colorful natural dyes. Int. J. Adv. Sci. Eng..

[23] Parra-Campos A, Ordóñez-Santos LE (2019). Natural pigment extraction optimization from coffee exocarp and its use as a natural dye in French meringue. Food Chem..

[24] Sullivan H, Wang B, Jiang L (2022). Investigation of tropical plant-based natural dyes combination and adsorption optimization for natural dye-sensitized solar cell. Environ. Prog. Sustain. Energy.

[25] Rajkumar S, Venkatraman MR, Balraju P, Suguna K, Pugazhendhi A (2022). Performance of simple green synthesized Ag incorporated TiO_2_ nanoparticles based photoanodes by doctor-blade coating as working electrodes for dye sensitized solar cells. Prog. Org. Coat..

[26] Lee KJ, Kim JH, Kim HS, Shin D, Yoo DW, Kim HJ (2012). A study on a solar simulator for dye sensitized solar cells. Int. J. Photoenergy.

[27] Vinaayak SB, Balasubramani V, Shkir M, Manthrammel MA, Sreedevi G (2022). Enhancing the performance of TiO_2_ based N-DSSC using dye extracted from Cladophora Columbiana, Ludwigia repens and mixed sensitizer. Opt. Mater..

[28] Swart HC, Kroon RE (2019). Ultraviolet and visible luminescence from bismuth doped materials. Opt. Mater. X.

[29] Rishi K, Rana N (2015). Particle size and shape analysis using image J with customized tool for segmentation of particles. Int. J. Comput. Sci. Commun. Netw..

[30] Salmani, I. A., Murtaza, T., Gupta, A., Khan, M. S. & Khan, MS. . Synthesis and structural properties of multiferroic Bi_0.95_Mg_0.05_FeO_3_. In *AIP Conference Proceedings 2018 May 8*, Vol. 1953, No. 1, p. 030132 (AIP Publishing LLC, 2018).

[31] Tanaka K, Hirao K, Soga N (1991). Synthesis of new amorphous oxides with ferromagnetic character in iron oxide-based systems. J. Appl. Phys..

[32] Mehra S, Bishnoi S, Jaiswal A, Jagadeeswararao M, Srivastava AK, Sharma SN, Vashishtha P (2020). A review on spectral converting nanomaterials as a photoanode layer in dye-sensitized solar cells with implementation in energy storage devices. Energy Storage.

[33] Ünlü B, Özacar M (2020). Effect of Cu and Mn amounts doped to TiO_2_ on the performance of DSSCs. Sol. Energy.

[34] Ahamad T, Aldalbahi A, Alshehri SM, Alotaibi S, Alzahly S, Wang ZB, Feng PX (2021). Enhanced photovoltaic performance of dye-sensitized solar cells based Ag_2_O doped BiFeO_3_ heterostructures. Sol. Energy.

[35] Taya SA, El-Agez TM, El-Ghamri HS, Abdel-Latif MS (2013). Dye-sensitized solar cells using fresh and dried natural dyes. Int. J. Mater. Sci. Appl..

[36] Pratiwi DD, Nurosyid F, Supriyanto A, Suryana R (2017). Performance improvement of dye-sensitized solar cells (DSSC) by using dyes mixture from chlorophyll and anthocyanin. J. Phys. Conf. Ser..

[37] Malik M, Iqbal MA, Choi JR, Pham PV (2022). 2D materials for efficient photodetection: Overview, mechanisms, performance and UV-IR range applications. Front. Chem..

